# Thrombin increases inflammatory cytokine and angiogenic growth factor secretion in human adipose cells *in vitro*

**DOI:** 10.1186/1476-9255-6-4

**Published:** 2009-03-06

**Authors:** Jennifer L Strande, Shane A Phillips

**Affiliations:** 1 Division of Cardiovascular Medicine, Medical College of Wisconsin, Milwaukee, WI, USA; 2 Department of Physical Therapy, University of Illinois, Chicago, IL, USA

## Abstract

**Background:**

Abdominal obesity is associated with pro-thrombotic and inflammatory states. Therefore, the purpose of this study was to examine the expression of thrombin receptors (PAR1 and PAR4) human adipose tissue and whether thrombin stimulates an inflammatory cytokine and growth factor profile in human adipose tissue.

**Methods:**

Human adipose tissue, isolated preadipocytes and differentiated adipocytes were used in this study. PAR1 and PAR4 mRNA and protein were detected by RT-PCR and immunoblot analysis in both adipose tissue and adipose microvessels. In separate studies, IL-1β, IL-6, MCP-1, TNF-α, IL-10, FGF-2, VEGF, and PDGF production were measured from adipose tissue (n = 5), adipocytes (n = 5), and preadipocytes (n = 3) supernatants with and without thrombin (1 or 10 U/ml; 24 hrs) treatment.

**Results:**

Thrombin increased cytokine secretion of IL-1β, IL-6, MCP-1 and TNF-α and growth factor secretion of VEGF from adipocytes along with MCP-1 and VEGF from preadipocytes. The direct thrombin inhibitor lepirudin given in conjunction with thrombin prevented the thrombin-mediated increase in cytokine and growth factor secretion.

**Conclusion:**

Here we show that thrombin PAR1 and PAR4 receptors are present and that thrombin stimulates inflammatory cytokine generation and growth factor release in human adipose tissue and cells *in vitro*. These data suggest that thrombin may represent a molecular link between obesity and associated inflammation.

## Background

Protease-Activated Receptors (PAR) belongs to a small family of seven transmembrane G protein-coupled receptors (GCPR) whose unique mechanism of action requires proteolytic cleavage of the N-terminus. This cleavage exposes a tethered ligand which then transactivates the receptor [1,2]. Serine proteases including thrombin and other coagulation factors such as Factor (F) Xa and the Tissue Factor (TF):FVIIa complex activate PAR1 and/or PAR4 [3,4]. PARs have been found to be expressed in a variety of tissues and cells including platelets, endothelial cells, leukocytes, and fibroblasts and modulate a variety of responses to thrombin including fibrosis, thrombosis, and inflammation [3].

PAR activation in non-adipose tissue induces the hallmarks of inflammation, including up-regulation of proinflammatory mediators and adhesion molecules, enhanced vascular permeability and leukocyte extravasation and infiltration [5,6]. Specifically, thrombin stimulates production of the proinflammatory cytokines interleukin (IL)-1, IL-6, and monocyte chemotactic protein (MCP)-1 from vascular endothelial cells [7-9]. Similarly, thrombin activation of monocytes increases the secretion of tumor necrosis factor (TNF)-α, IL-1β, IL-6, and MCP-1 [9-11]. Thrombin also stimulates angiogeneis and contributes to the increased expression of angiogenic growth factors including fibroblast growth factor (FGF)-2, platelet derived growth factor (PDGF) and vascular endothelial growth factor (VEGF) [7,12-14]. The inflammatory and angiogenic properties of thrombin have important roles in the pathogenesis of atherosclerosis [15]. However, it is unknown what role these receptors play in modulating the inflammatory response associated with adipose tissue accumulation.

Abdominal adipose mass is associated with the metabolic syndrome representing a compilation of abnormalities including insulin resistance, hyperlipidemia, hypertension, and atherosclerosis leading to cardiovascular disease [16]. Excess and/or dysfunctional adipose tissue, particularly visceral adipose tissue is associated with a chronic low-grade systemic inflammation. Hypercoagulation is also associated with obesity and the metabolic syndrome [17,18] such that the levels of PAR activating proteases (e.g. thrombin, TF, FVIIa, and FXa) are elevated [18-22]. However, the contribution of coagulation factors such as thrombin to adipose-mediated inflammation is unknown.

In this study, we tested the hypothesis that thrombin receptors are expressed in adipose tissue and that thrombin modulates inflammatory cytokine and angiogenic growth factor release in human adipose tissue and cells.

## Methods

### Subjects

Discarded and de-identified visceral adipose samples were obtained from 16 patients at the time of abdominal surgery. The patients ages ranged from 34 – 64 years old with one patient > 80 years old and BMI ranged from 19.5 – 33.5 (average 25.9). Since the tissues used were otherwise discarded and de-identified during the surgical procedure we were unable to ascertain the specific criteria used to make the diagnosis or quantify the severity and duration of medical therapy, if any. Most surgeries were done for bowel resections, liver resections or exploratory laparoscopies as a result of trauma. None of the patients had cardiovascular disease. All studies were approved by the institutional IRB committee. Adipose tissue was used for acute adipose tissue culture and isolation of the stromal-vascular (S-V) fraction.

### Materials

All cell cultureware were purchased from Fisher Scientific (Norcross, GA). Bacterial collagenase *Clostridium histolyticum *(type 1) was obtained from Worthington Biochemicals (Lakewood, NJ). 1× RBC Lysis Buffer was obtained from eBioscience (San Diego, CA). Media and supplements were purchased from PromoCell (Heidelberg, Germany). Fatty-Acid Free (FAF)-bovine serum albumin (BSA) and human thrombin was obtained from Sigma (St. Louis, MO) along with all other chemicals and reagents unless otherwise stated.

### Human Adipose Tissue and Cell Culture

Human adipose tissue pieces contain the different cell types including fibroblasts, endothelial and adipose cells and permit long-term culture [23]. Thus, primary culture of adipose tissue pieces preserves paracrine interaction among cells that can influence adipocyte metabolism and offers the unique possibility to study this interaction in culture. Preparation of adipose tissue cultures was performed as previously described [23]. Briefly, adipose tissue was minced into small pieces (2–4 mm^2^) using scissors under aseptic conditions. The minced tissue was washed twice in HBSS/2% FAF-BSA by centrifuging at 450 g × 5 min followed by pouring the minced tissue through a 200 μm nylon mesh and rinsing with 300 ml sterile PBS. The explants (0.5 mg/ml) were incubated for the indicated times in suspension culture under aseptic conditions at 37°C and 5% (v/v) CO_2_.

The S-V cell fraction was isolated as previously described [24]. In brief, minced adipose tissue (1–3 grams) was washed twice and then placed in digestion solution (200 U/ml collagenase, 1% FAF-BSA, and PBS to 10 ml) in a shaker (200 rpm) at 37°C for 1 hr. The collagenase digest was separated from undigested tissue, adipocytes and lipids by centrifugation at 850 g × 5 min. The pellet containing the S-V cell fraction was then resuspended in 10 ml 1× RBC Lysis Buffer and incubated for 10 min at room temperature. The sample was centrifuged again and the cell pellet was resuspended in PromoCell Preadipocyte Growth Medium. The cells were cultured on non-coated plates to limit adherence of endothelium thereby enriching the environment for preadipocytes. Preadipocytes were differentiated at passage 3 by adding PromoCell Adipocyte Differentiation Medium for 72 hrs and then further culturing the cells in PromoCell Adipocyte Growth Medium until differentiation was complete (12–14 days). The life-span of unstimulated macrophages in culture is 5–6 days [25]. At the time of the experiments more than 70% of the primary culture cells were filled with multiple lipid droplets. The lipid accumulations in the differentiated adipocytes were assessed through staining of the neutral fats and cholesterol esters using Oil Red O dye (Sigma). In brief, the differentiated adipocytes were fixed in 4% formaldehyde for 10 min, and then stained for 30 min with the Oil Red O dye dissolved in isopropanol. The stained adipocytes were washed with PBS.

### Protein Extraction for Immunoblot Analysis

Adipose tissue was dissected free of microvessels and fibrous tissue. The dissected microvessels and adipose tissue were put into separate tubes and flash frozen in liquid nitrogen. Ice-cold RIPA buffer (50 mM Tris-HCL, pH 7.5, 1% Nonidet P-40, 0.1 mM EDTA, 0.1 mM EGTA, 0.1% SDS, 0.1% deoxycholic acid) was added and the tissue was homogenized for 15 strokes and centrifuged at 10,000 × *g *at 4°C for 10 min. Protein concentration was measured by the bicinchoninic acid protein assay.

### Immunoblot Analysis

Fifty micrograms of protein from each sample was mixed with 10× reducing SDS loading buffer, boiled for 5 minutes, run on a 10% polyacrylamide SDS gel and transferred to a PVDF membrane. After the membrane was blocked for 1 hr at room temperature with 5% non-fat milk in TBS with 0.1% Tween-20 (TBS-T), it was incubated with a 1:200 dilution of the primary antibody (Anti-Thrombin Receptor (PAR1), Cat No. 611523BD, Biosciences, San Jose, CA or PAR4 (C-20), Cat No. sc-8464, Santa Cruz, CA) in TBS containing 0.1% Tween-20, 0.5% dry milk and 0.1% sodium azide overnight at 4°C. The blots were then incubated with the secondary antibody for 1 hr at room temperature, followed with enhanced chemiluminescence solution (Amersham Life Science, Arlington Heights, IL) and then exposed to film for 1 minute. Cell lysates from the A549 cell line (ProSci Incorporated, Poway, CA) was used for a positive control.

### RT-PCR

Forward and reverse primers used for amplifying human PAR1 and PAR4 were prepared commercially, based on published data (accession number NM_001992 and NM_003950): PAR1 sense 5'-CCGCCTGCTTCAGTCTGT-3', antisense 5'-TCATCCTCCCAAAATGGTTC-3' (PCR product 151 bp) and PAR4 sense 5'-CAGAGCAGCCTGAGTGCAG-3' and antisense 5'-CAGGGTGTCACTGTCATTGG-3' (PCR product 207). The conditions for amplification were 95°C for 2 min for one cycle, 95°C for 30 s, 62°C for 30 s, 72°C for 60 s for 30 cycles and 72°C for 1 min for 1 cycle. Two microliters of cDNA was used in each reaction along with iTaq DNA Polymerase and dNTP mix (Bio-Rad, Hercules, CA). Electrophoresis was conducted on a 2% agarose gel stained with SYBR green.

### Thrombin Stimulation of Adipose Tissue, Adipocytes and, Preadipocytes

Confluent cells were washed twice with PBS and then incubated with Starvation Media (DMEM/F12 with d-Biotin 8 μg/ml and bovine insulin (0.5 μg/ml). After 6 hours, the media was replaced with fresh Starvation Media containing PBS, IBMX (100 μM), or thrombin (1 or 10 U/ml) or thrombin (10 U/ml) and lepirudin (100 ATU/ml). Minced adipose tissue samples were cultured in Assay Buffer (Zen-Bio, Inc, Research Triangle Park, NC) with PBS, thrombin (1 U/ml) or thrombin (10 U/ml) with or without lepirudin (100 ATU/ml). After 24 hours, conditioned medium was collected and stored at -80°C until analysis. Interleukin-1β, IL-6, TNF-α, MCP-1, IL-10, FGF-2, PDGF-BB, and VEGF were determined in duplicate by a human cytokine Bioplex assay using a custom kit from BioRad (Hercules, CA).

### Statistical Analysis

Data are reported as mean ± SEM. Statistical analyses were performed by the Student's *t *test for paired values and a one-way Analysis of Variance (ANOVA) for differences between treatment groups. If significant, a Newman-Keuls multiple comparison test was used to perform pair wise comparisons. Data were considered significant at a *p *< 0.05. Statistics were performed using WINKS SDA Software (Texasoft, Cedar Hill, TX).

## Results

### PAR1 and PAR4 Expression in Adipose Tissue

**Figure 1:**
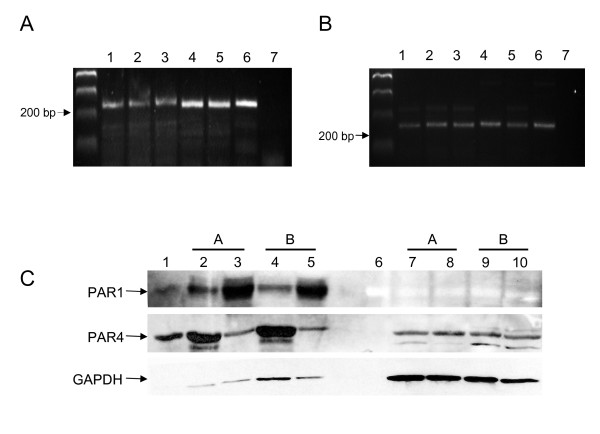
**PAR1 and PAR4 are present in adipose tissue**. A) PAR1 mRNA amplified from cDNA from adipose tissue from 5 individuals (lanes 1–5). The positive control (lane 6) contains cDNA from a human artery and the negative control (lane 7) contains PAR1 specific primers but no cDNA. B) PAR4 mRNA amplified from cDNA from adipose tissue from 5 individuals (lanes 1–5). The positive control (lane 6) contains cDNA from a human artery and the negative control (lane 7) contains PAR4 specific primers but no cDNA. C) Representative immunoblots of tissue or cells from 2 individuals (A and B). PAR1 and PAR4 protein detected in adipose tissue (lanes 2 and 4) and adipose microvessels (lanes 3 and 4) but only PAR4 is detected in Preadipocytes (lanes 7 and 9) and differentiated adipocytes (lanes 8 and 10). Lane 1 is a positive control lysate. Lane 6 contains the molecular weight marker. A total of 6 groups (whole tissue and isolated preadipocytes and differentiated adipocytes) were analyzed for PAR1 and PAR4.

cDNA reverse transcribed from adipose tissue mRNA from 5 individuals was amplified using primers specific for the human PAR1 and PAR4 sequences. PAR1 mRNA is constitutively expressed in the human adipose tissue (Figure 1A, lanes 1–5). PAR4 mRNA is also detected in human adipose tissue (Figure 1B, lanes 1–5). Human adipose tissue from 6 individuals was dissected free of microvessel and protein was extracted from both the adipose tissue and microvessels and used for immunoblotting for PAR1 and PAR4. A representative immunoblot of tissue from 2 individuals is shown in Figure 1C. Both PAR1 and PAR4 were detected in both adipose tissue and the microvessels. When equalized for protein loading, PAR1 protein was higher in the adipose vasculature. While PAR4 is more prominent in adipose tissue than microvessels, to further localize the cell type expressing PAR1 or PAR4, adipose tissue tissues from the same 6 individuals were digested and the S-V fraction was isolated. The S-V fraction was split and cultured as preadipocytes or differentiated into adipocytes cells. A representative immunoblot of the cell lysates from adipocytes and preadipocytes from 2 individuals is shown in Figure 1C. The presence of PAR4, but not PAR1 is detected.

### Effect of Thrombin on Pro-Inflammatory Cytokine Secretion

**Figure 2:**
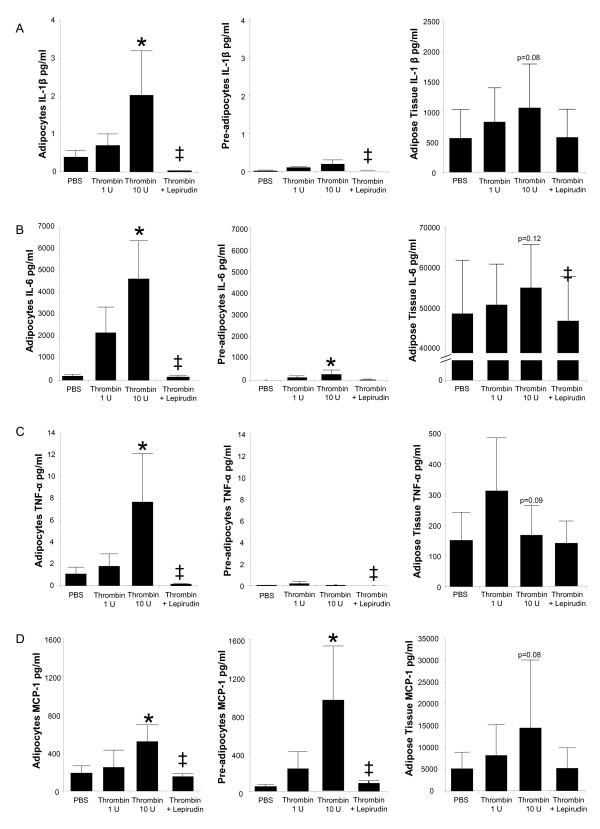
**Thrombin stimulates inflammatory cytokine secretion predominately from primary cultures of newly differentiated human adipocytes**. Cultures of adipose tissue (*n *= 5), newly differentiated adipocytes (*n *= 5) or preadipocytes (*n *= 3) were stimulated with thrombin (1 U/ml) or thrombin (10 U/ml) in the presence or absence of a thrombin inhibitor (lepirudin) for 24 hours. PBS served as the negative control. The following cytokines were measured from the supernatants of confluent cultures. A) IL-1β; B) IL-6; C) TNF-α; D) MCP-1. Means ± SEM,. *P < 0.05 thrombin vs. PBS. ± P < 0.05 thrombin + lepirudin vs. thrombin.

The effect of thrombin on pro-inflammatory cytokine secretion was assessed using human adipose tissue, adipocytes, and preadipocytes cultures (Table 1 and Figure 2). Both 1 U/ml and 10 U/ml thrombin was used to stimulate the cultures; however the 1 U/ml thrombin concentration failed to induce a significant cytokine secretion. Thrombin (10 U/ml) increased the secretion from differentiated adipocytes but not preadipocytes or adipose tissue (Figure 2A). In addition, the direct thrombin inhibitor, lepirudin completely abolished the IL-β production by thrombin (10 U/ml) in these cultures. Thrombin (10 U/ml) also enhanced IL-6 release in adipocytes and preadipoctyes (Figure 2B). The basal secretion from preadipocytes was lower than the basal secretion from adipocytes. Lepirudin completely abolished thrombin-mediated secretion of IL-6 from adipose tissue, adipocytes and preadipocytes. Thrombin (10 U/ml) stimulation of adipocyte cultures increased TNF-α secretion and MCP-1 secretion, and increased preadipocyte MCP-1 release (Figure 2C and Figure 2D). Lepirudin abolished these responses.

**Table 1:** Secretion of cytokines and growth hormones after stimulation of adipose tissue, differentiated adipocytes, or pre-adipocytes with thrombin.

		**Adipocytes** *n *= 5	**Preadipocytes** *n *= 3	**Adipose Tissue** *n *= 5
**IL-1β**	24 h secretion	2.0 ± 1^a^	0.2 ± 0.1	1062 ± 721
	% induction	616 ± 341	717 ± 453	195 ± 74
**IL-6**	24 h secretion	4638 ± 1756^a^	272 ± 201^a^	54682 ± 10734
	% induction	3100 ± 1400	2274 ± 1041	22 ± 13
**TNF-α**	24 h secretion	7.7 ± 4.5^a^	0.5 ± 0.5	168 ± 97
	% induction	906 ± 522	25 ± 20	44 ± 30
**MCP-1**	24 h secretion	521 ± 167^a^	951 ± 577^a^	14421 ± 6996
	% induction	520 ± 370	2451 ± 842	144 ± 64
**FGF-2**	24 h secretion	4.8 ± 2.1^a^	1.3 ± 1.2	287 ± 104
	% induction	195 ± 106	52 ± 44	202 ± 140
**PDGF**	24 h secretion	1.4 ± 0.9^a^	0.7 ± 0.3^a^	170 ± 144
	% induction	198 ± 28	635 ± 131	77 ± 52
**VEGF**	24 h secretion	4662 ± 1884^a^	13465 ± 10177^a^	3783 ± 609^a^
	% induction	401 ± 96	634 ± 131	134 ± 87

Differentiated Adipocytes, Preadipocytes or Adipose Tissue cultures were incubated for 24 h in the presence of thrombin (10 U/ml). Cytokine and growth factor release to the medium is in pg/ml for adipocytes and preadipocyte cultures and pg/ml/g for adipose tissue cultures. % induction is the average of the individual % cytokine/growth factor induction from each sample. The values are shown as the mean ± SEM. ^a^P < 0.05 thrombin vs. control.

### Effect of Thrombin on Anti-Inflammatory Cytokine Secretion

Secretion of IL-10 from thrombin (1 and 10 U/ml) stimulated adipose tissue, adipocytes and preadipocytes was below the detection limit (data not shown).

### The Effect of Thrombin on Growth Factor Secretion

**Figure 3:**
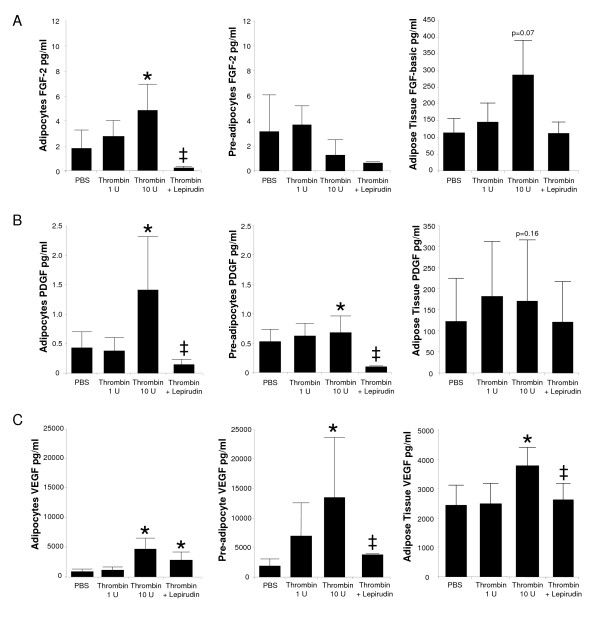
**Thrombin stimulates angiogenic growth hormone secretion predominately from primary cultures of newly differentiated human adipocytes**. Cultures of adipose tissue (*n *= 5), newly differentiated adipocytes (*n *= 5) or preadipocytes (*n *= 3) were stimulated with thrombin (1 U/ml) or thrombin (10 U/ml) in the presence or absence of a thrombin inhibitor (lepirudin) for 24 hours. PBS served as the negative control. The following growth factors were measured from the supernatants of the cultures. A) FGF-2; B) PDGF and; C) VEGF. Means ± SEM *P < 0.05 thrombin vs. PBS. ± P < 0.05 thrombin + lepirudin vs. thrombin.

Human adipose tissue explants, adipocytes, and preadipocytes cultures were also used to assess the effects of thrombin on angiogenic growth hormone secretion (Table 1 and Figure 3). Again 1 U/ml and 10 U/ml thrombin concentrations were used to stimulate the cells, yet no significant stimulation was found with the lower concentration. Thrombin (10 U/ml) increased FGF-2 secretion from adipocytes and PDGF secretion from adipocytes and preadipocytes (Figure 3A and Figure 3B). The addition of lepirudin reversed these effects. Additionally, thrombin (10 U/ml) enhanced VEGF secretion in adipose tissue and adipocytes and preadipocyte cultures (Figure 3C). Again this secretion was diminished by lepirudin.

## Discussion

There are three novel findings of this study. First, our results indicate that thrombin PAR1 and PAR4 receptors are present in adipose tissue. Second, thrombin stimulated secretion of pro-inflammatory (IL-1β, IL-6, MCP-1, and TNF-α) cytokines in human adipocytes, an effect that was blocked by the thrombin inactivating peptide lepirudin. Third, thrombin induced secretion of the major angiogenic growth hormones, FGF-2 PDGF, and VEGF from adipocytes and PDGF and VEGF from preadipocytes. Again, these effects were blocked by lepirudin. Taken together these data suggest that thrombin modulates the inflammatory and angiogenic status of adipose tissue.

### Thrombin Receptors in Adipose Tissue

Our studies indicate that PAR1 is expressed primarily in the microvasculature of the adipose tissue rather than in the adipocytes. In contrast, PAR4 is expressed in the non-vascular cells in human adipose tissue. These findings are similar to other reports indicating PAR1 is the predominant thrombin receptor in human vasculature [26]. However, this is the first study to show that PAR4 is preferentially expressed in human preadipocytes and adipocytes suggesting that the thrombin-mediated responses of these cells are mediated by PAR4 activation.

### Characterization of Cultures

The various cell types within adipose tissue including adipocytes, preadipocytes, fibroblasts, macrophages, and vascular cells are able to contribute to the secretion of cytokines and growth factors [27,28]. Each cell type contributes to the cytokine footprint under different conditions and stimuli. Therefore, we stimulated both isolated preadipocytes and differentiated adipocytes (from the same tissue) with thrombin to determine which cell type contributes to the secretion of cytokines and growth factors. We have shown that thrombin preferentially stimulates the secretion of IL-1β, IL-6, and TNF-α from differentiated adipocytes. It has previously been shown, that unstimlutated non-adipocytes contribute the majority of basal IL-1β, IL-6, and TNF-α secretion [28]. Therefore, either thrombin is able to shift the balance in cytokine secretion from non-adipoctyes to adipocytes or this finding may be attributed to the differences in experimental design. In the work by Fain *et al*., the cytokine levels were measured within 48 hours of adipocytes and S-V cells isolation when macrophages and endothelial cell numbers are relatively high in the S-V fraction [28]. However, it is unlikely that macrophages were involved in the cytokine production in our cell cultures for several reasons. First, macrophages have a short-life span in culture making survival unlikely in our cultures where preadipocytes and differentiated adipocytes were stimulated after ~3 weeks of culture conditions [25]. Second, isolated S-V fractions were divided in order to keep half the cells in an undifferentiated state while the other half was differentiated. Given that conditions and time course were similar at the time of study between fractions, it is likely that macrophage quantity was similar. If there was macrophage survival under both conditions, a similar secretion profile would be expected.

We also found that thrombin mediated similar levels of MCP-1 secretion in adipocytes and preadipocytes. This is consistent with other studies showing that cells from the S-V fraction or preadipocytes are a major source of MCP-1 secretion even though adipocytes also secrete MCP-1 [29-31]. The increase in MCP-1 secretion from our adipocytes cultures may be from the adipocytes themselves or from the number of preadipocytes cells in adipocytes cultures that did not undergo differentiation.

Finally, we have shown that thrombin increases the secretion of VEGF from adipocytes and preadipocytes cultures. However, VEGF was higher in preadipocytes than adipocytes suggesting VEGF may be preferentially secreted from these cell types. This is consistent with other studies indicating adipocytes have VEGF secretion capabilities in response to neurohumoral factors such as insulin but that non-adipocytes are the main secretors of VEGF [28-32]. Taken together with other studies that show preadipocytes induces VEGF release in response to adipocytes differentiation [33], our studies support the conclusion that both cell types contributes to VEGF secretion. Thrombin is a known stimulus for angiogenesis; therefore, thrombin-mediated VEGF from preadipocytes and adipocytes may be important for the angiogenesis that occurs during adipogenesis and adipose expansion. Lepirudin, a direct thrombin inhibitor abolished the thrombin-mediated increase of IL-1β, IL-6, MCP-1 and TNF-α secretion along with VEGF suggesting that cytokine and angiogenic growth hormone secretion (Figure 2 and Figure 3) in our studies was related to the interaction of thrombin with its receptor, PAR4, in adipocytes.

Even though there was a trend towards increased secretion of IL-1β, IL-6, MCP-1 and TNF-α in thrombin stimulated adipose tissue, it did not reach significance. However, the baseline production in these tissues was higher than our cell culture measurements (adipocytes and preadipocytes) suggesting that the contribution of cytokine production from the non-adipocyte components of adipose tissue (i.e. endothelial cell and macrophages) are additive. The contribution of cytokine production from adipocytes should not be dismissed since 1) cytokine secretion even at low levels may be physiologically relevant and 2) most cytokines have autorcrine and paracrine signalling such that the localized effects of these compounds for vascular and adipocyte function. Thrombin is known to stimulate IL-1β and TNF-α secretion from endothelial cells, monocytes, and fibroblasts which are residential cells within adipose tissue supporting previous reports that these cell types contribute to cytokine production in human adipose tissue [9,11].

### Inflammatory Cytokines and Adipose Tissue

Several inflammatory cytokines including TNF-α, IL-1β, IL-6, and MCP-1 are released from adipocytes. These factors are also increased in obesity and contribute to insulin resistance and atherosclerosis [34,35]. Thrombin activation of adipose cells may contribute to the circulating inflammatory cytokines and insulin resistance found in obese individuals. It is also possible that the inflammatory phenotype induced by thrombin in obesity promotes vascular inflammation and endothelial dysfunction thereby advancing atherosclerosis. Thrombin activates NF-κB thereby up-regulating a variety of cytokines in other tissues and cells [3], a mechanism that may signal thrombin-mediated release of inflammatory cytokines from adipose tissue. Our results support the conclusion that thrombin may contribute to the inflammatory state by increasing IL-1β, IL-6, MCP-1, TNF-α in adipose tissue.

### Angiogenic Growth Factors and Adipose Tissue

Angiogenesis plays an important role in atherosclerosis and adipose tissue expansion [36]. The specific role of VEGF in adipose angiogenesis and whether the enhanced levels of coagulation factors associated with obesity increase VEGF release by adipose tissue and thus contribute to angiogenesis is unknown. However, we have shown that thrombin increases the secretion of growth hormones (FGF-2, PDGF, and VEGF) from adipocytes and VEFG from adipose tissue which suggests that thrombin may contribute to angiogenic process in visceral adipose tissue. Future studies are needed to explore the contribution of adipocyte derived growth factors to preadipocyte differentiation and angiogenesis in obesity.

### Study Limitations

There are several limitations of this study. First, we did not measure thrombin-mediated cytokine release from a homogenous cell line where variability may be minimized. On the other hand homogenous cell cultures make it difficult to mimic the complexity and chronicity of disease processes such as obesity even though we might expect less variation. Even though the cell culture conditions were kept constant and grown to a similar maturity, there was a lot of variability in the basal secretion of cytokines and growth factors between samples indicating that the variability is most likely due to the genetic heterogeneity of the human tissues. Despite this limitation, none of our patients had coagulation disorders, heart disease, or morbid obesity that may have confounded our results. Second, tissue was obtained from discarded specimens so it was impossible to obtain comprehensive clinical data on our patient population making it difficult to correct for the physiological confounding effects of our subject population such as body fat distribution and severity of disease. However, all subjects had a BMI<34 and no cardiovascular risk factors, thereby eliminating most the confounding effects of overt obesity and cardiovascular disease on cytokine production from fat. Despite this recruitment strategy, this study demonstrates an increase in thrombin-mediated cytokine secretion from which future studies will focus on the effects of age, sex, and body fat distribution on the physiological responses of adipose to thrombin. Third, our study was limited to the effect of thrombin on cytokine and angiogenic growth factor release from abdominal adipose. Since, other adipose depots may respond differently, future studies may focus on the inflammatory profile of other adipose depots that may contribute to cardiovascular disease (e.g. pericardial adipose). Fourth, our patient population was not morbidly obese. This was an unavoidable limitation of our study design using discarded and de-identified surgical samples. While most of the cytokines measured in this study are elevated in obesity, it is unknown whether thrombin induced cytokine release is dependent on the patient's adipose tissue distribution profile making it difficult to discern the contribution of thrombin mediated cytokines to obesity and other associated diseases (i.e. cardiovascular disease). Future studies will determine relationships between the thrombin-mediated inflammatory response, PAR 4 expression in isolated adipose tissue cell types, and body adipose mass.

## Conclusion

This study demonstrates the presence of PAR1 and PAR4 in adipose tissue and PAR4 in isolated populations of adipocytes and preadipocytes. Thrombin stimulation of adipocytes and preadipocytes increases pro-inflammatory cytokines (IL-1β, IL-6, TNF-α, MCP-1) along with the angiogenic growth factor (VEGF). Therefore, thrombin may contribute to the inflammatory state associated with visceral adipose tissue.

## Abbreviations

ANOVA: Analysis of variance; FGF-2: Fibroblast growth factor; GCPR: G protein-coupled receptors; IL-6: Interleukin-6; IL-1: Interleukin-1; MCP-1: Monocyte chemotactic protein; PDGF: Platelet derived growth factor; S-V: Stromal-vascular fraction; TNF-α: Tumor necrosis factor; VEGF: vascular endothelial growth factor

## Competing interests

The authors declare that they have no competing interests.

## Authors' contributions

JLS conceived the study, participated in its design and carried out all experiments and analysis and drafted the manuscript. SAP participated in the study design and coordination and helped to draft the manuscript. Both authors have given final approval of the version to be published.
